# Physiological, Transcriptomic, and Metabolic Responses of *Ginkgo biloba* L. to Drought, Salt, and Heat Stresses

**DOI:** 10.3390/biom10121635

**Published:** 2020-12-03

**Authors:** Bang Chang, Kaibiao Ma, Zhaogeng Lu, Jinkai Lu, Jiawen Cui, Li Wang, Biao Jin

**Affiliations:** College of Horticulture and Plant Protection, Yangzhou University, Yangzhou 225009, China; MX120180603@yzu.edu.cn (B.C.); makaibiao@126.com (K.M.); d160068@yzu.edu.cn (Z.L.); DX120200123@yzu.edu.cn (J.L.); DX120180104@yzu.edu.cn (J.C.); liwang@yzu.edu.cn (L.W.)

**Keywords:** *Ginkgo biloba* L., drought stress, salt stress, heat stress, transcriptome, miRNA, metabolome

## Abstract

*Ginkgo biloba* L. is highly adaptable and resistant to a range of abiotic stressors, allowing its growth in various environments. However, it is unclear how *G. biloba* responds to common environmental stresses. We explored the physiological, transcriptomic, and metabolic responses of *G. biloba* to short-term drought, salt, and heat stresses. Proline, H_2_O_2_, and ABA contents, along with CAT activity, increased under all three types of stress. SOD activity increased under salt and heat stresses, while soluble protein and IAA contents decreased under drought and salt stresses. With respect to metabolites, D-glyceric acid increased in response to drought and salt stresses, whereas isomaltose 1, oxalamide, and threonine 2 increased under drought. Piceatannol 2,4-hydroxybutyrate and 1,3-diaminopropane increased under salt stress, whereas 4-aminobutyric acid 1 and galactonic acid increased in response to heat stress. Genes regulating nitrogen assimilation were upregulated only under drought, while the GRAS gene was upregulated under all three types of stressors. ARF genes were downregulated under heat stress, whereas genes encoding HSF and SPL were upregulated. Additionally, we predicted that miR156, miR160, miR172, and their target genes participate in stress responses. Our study provides valuable data for studying the multilevel response to drought, salinity, and heat in *G. biloba*.

## 1. Introduction

Plants frequently experience adverse growth conditions, including drought, salinity, and extreme temperatures. These stresses limit development primarily by affecting physiological and biochemical processes and cellular homeostasis. Drought and salinity impose osmotic stress, resulting in turgor loss in cells, and salinity may also induce an imbalance in cellular ion homeostasis [1,2]. Heat stress may reduce cell water content. In addition, environmental stress may lead to disorganization of cell membranes and excess reactive oxygen species (ROS) production, resulting in oxidative damage [3,4].

Plants have a remarkable capacity to cope with a variety of environmental stresses. Survival under environmental stress requires the integration of adaptive metabolic, physiological, and molecular responses [5,6]. For example, plants may control water flux and cellular osmotic adjustment via stomatal closure, activation of respiration, and biosynthesis of osmoprotectants to resist drought, salt, and heat stresses [1]. In addition, a range of protein kinases and transcription factors involved in stress signaling serve to regulate ionic and osmotic homeostasis [5]. Under salt stress, plants sense ionic (Na^+^) toxicity on the surface of the plasma membrane of cells, where SnRK3 SOS2-like protein kinases interact with SOS2-like protein kinases to activate the Na^+^/H^+^ antiporter SOS1 via the SOS pathway, thereby maintaining Na^+^ homeostasis in cells [2]. Drought may trigger production of abscisic acid (ABA), which in turn stimulates stomatal closure and expression of stress-related genes. For example, the ABA-activated SnRK2 protein kinase regulates stomatal closure [4,5]. In addition, Dehydration-responsive element binding protein 1 and 2 (DREB2) are induced by dehydration and may activate other genes involved in drought tolerance [5,7]. Heat stress induces heat shock proteins (HSPs, e.g., HSP70, HSP90, and HSP100) and complex transcriptional networks, including HEAT SHOCK TRANSCRIPTION FACTOR A1s (HSFA1s) and DREB2A. Among these, HSFA1s is the master regulator of the heat stress response, directly regulating important transcription factors such as DREB2A, HSFA2, and HSFBs [3]. Plants thus respond to different environmental stressors via a variety of pathways; however, different transcription factors may be involved in the responses to different types of stress. While such responses have been evaluated extensively and intensively in model species, studies on gymnosperms are lacking.

*Ginkgo biloba* L., sometimes referred to as a “living fossil”, is a relict gymnosperm species [8] that has been introduced in many countries [9]. *G. biloba* exhibits a high degree of environmental adaptability and resistance to pathogens and consequently has a very long lifespan [10]. Studies of abiotic stress in *G. biloba* have focused mainly on leaf morphology and observations of anatomical structures, osmotic regulators, secondary metabolites, and photosynthetic capacity [11,12,13]. The rapid development of omics technology in recent years has facilitated research on the molecular responses of *G. biloba* to abiotic stress. The v-myb avian myeloblastosis viral oncogene homolog transcription factor family plays an important role in development, defense, and flavonoid biosynthesis in *G. biloba* [14]. Cao et al. [15] isolated three HSP genes (*GbHSP16.8*, *GbHSP17*, and *GbHSP70*) from *G. biloba* leaves subject to cold stress. In addition, the flavonoid biosynthesis-related genes *GbPALs* and *GbFLSs* were found to be significantly upregulated under salt stress [16]. However, few reports have examined stress resistance mechanisms at the transcriptional, post-transcriptional, and metabolite levels. Therefore, we hypothesize that there are different biomolecules involved in the responses to different stresses in *G. biloba*.

In this study, we analyzed the physiological, transcriptomic, and metabolic responses of *G. biloba* leaves to drought, salt, and heat stresses at the transcriptional and post-transcriptional levels. We analyzed soluble sugar, soluble protein, proline, ROS, superoxide dismutase (SOD), catalase (CAT), ABA, and auxin (IAA) and identified key transcription factors and other proteins involved in the responses to the three types of stress. In addition, we identified several miRNAs and target genes implicated in stress responses. Finally, we constructed the mode pattern of the responses to the three types of stress. The identification of these physiological and biochemical indicators and genes, transcript factors, and miRNAs in response to stresses, provides valuable insights for elucidating the response mechanism to drought, salt, and heat stresses in gymnosperms.

## 2. Materials and Methods

### 2.1. Plant Growth and Treatments

We used 2-year-old seedlings in the stress experiments, as 1-year-old plants do not have mature woody stems. Seeds were collected from a single parent plant at the experimental base at Yangzhou University (32°20′ N, 119°30′ E). Seeds were sown in 650 mm × 650 mm × 750 mm nursery pots in a 1:1:1 *v*/*v*/*v* mixture of soil, vermiculite, and perlite. Following germination, we thinned seedlings to three per pot. *G. biloba* seedlings were grown in the natural environment under full sun. The potted seedlings were placed in the growth chambers (day 25 °C, 16 h/night 18 °C, 8 h) a week before experiments. Next, all the stress treatments were conducted in the growth chambers. Irrigation is usually carried out when the weight of the pots significantly decreased. Distilled water was added to trays for soaking pots. 2 h later, the surplus water was poured away. We irrigated the tray with 1/2 MS nutrient solution once per month after the leaf expanded (during the growing period). After leaf expansion in June of the following year, we selected seedlings exhibiting robust growth and a uniform size for use in the stress treatments, and the seedling sizes were about 18–22 cm high and 8–12 cm wide (Figure S1). All seedlings were divided into four groups with five pots per group, and each group contained at least 15 seedlings. One group was used as CK, and the other three were subjected to D (drought), S (salt), and H (heat) treatments. Based on preliminary experiments, we used 20% PEG6000 to simulate D, a 200 mmol/L NaCl solution to simulate S, and a temperature of 40 °C to simulate H. Infrared thermography (FLIR E8) indicated that the leaf temperatures had increased significantly after 24 h of exposure to the stress treatments. Based on these observations, a 24-h treatment period was chosen for the experiment.

Pots used for D treatment were irrigated and soaked in a 20% PEG6000 solution for 24 h. For S treatment, pots were irrigated and soaked in 200 mmol/L NaCl solution for 24 h. In the H treatment, pots were subjected to 40 °C in growth chambers for 24 h. Since the top of the leaves were too young to fully expand, and older leaves at the base may be insensitive to respond to stresses, thus we selected leaves in the middle of the plants with a full expand status. Considering the similar growth status and the size of sampled plants, we further selected three seedlings with plenty of leaves from the 15 seedlings as the samples for transcriptomic and metabolic analyses. We did five biological repetitions of the metabolic analysis, and selected another two seedlings (a total of five seedlings) as the samples for metabolic analysis. We sampled the leaves following the treatment applications, and the leaves were rinsed in distilled water, dried with paper towels, and immediately snap-frozen in liquid nitrogen. Samples were stored at −80 °C prior to analysis. The samples of physiological analyses were from the same batch samples used for transcriptomic and metabolic analyses.

### 2.2. RNA Extraction, Sequencing, and Analyses

Total RNA was isolated from the leaves of three biological replicates from each treatment for use in RNA extraction and sequencing. Total RNA was extracted using the Mini BEST Plant RNA Extraction Kit (TaKaRa Bio Inc., Beijing, China) and treated with a genomic DNA Eraser (TaKaRa Bio Inc.) to reduce or eliminate DNA contamination. RNA integrity was assessed using the RNA Nano 6000 Assay Kit and the Bioanalyzer 2100 system (Agilent Technologies, Santa Clara, CA, USA). A total of 12 RNA-seq libraries were prepared using the NEBNext Ultra RNA Library Prep Kit for Illumina (NEB, Ipswich, MA, USA). Illumina-based sequencing was conducted on the HiSeq 2500 platform (Illumina Inc., San Diego, CA, USA). Following removal of reads containing adapters or ploy-N and low-quality reads, filtered reads were mapped to the *Arabidopsis thaliana* genome (TAIR 10) using TopHat2. We then calculated reads per kilobase of transcript per million mapped reads for each gene, based on gene length and the number of reads mapped to each gene. Differential expression analysis was performed using the DESeq package (1.18.0) in R. A fold change ≥1.5 and an adjusted *p*-value <0.05 were used as thresholds to evaluate the significance of differences in transcript levels. Sequenced data were deposited in the Genome Sequence Archive (GSA) database under accession number CRA003339.

### 2.3. Metabolomic Analysis

As the internal standard, 0.48 mL of methanol: water (3:1, *v*/*v*) and 24 μL of adonitol (1 mg/mL stock in dH_2_O) were added to 0.06 g of each sample in a 2 mL Eppendorf tube. The mixture was homogenized in a ball mill for 4 min at 50 Hz, followed by sonication for 2 × 5 min, while incubating in ice water. After centrifugation at 13,000× *g* at 4 °C for 15 min, 350 μL of supernatant was transferred into fresh 2 mL GC–MS glass vials. After drying the samples with a vacuum concentrator, we added 80 μL methoxyamine hydrochloride (20 mg/mL in pyridine) to each sample and incubated at 80 °C for 30 min. We then added 100 μL of BSTFA regent (1% TMCS, *v*/*v*) and incubated at 70 °C for 1.5 h, mixing the mixture well prior to GC–TOF–MS analysis.

GC–TOF–MS analysis was performed using the 7890A gas chromatograph system coupled with the Pegasus HT time-of-flight mass spectrometer (Agilent Technologies). The system used a DB-5MS capillary column coated with 5% diphenyl cross-linked with 95% dimethylpolysiloxane (30 m × 250 μm inner diameter, 0.25 μm film thickness; J&W Scientific, Folsom, CA, USA). A 1 μL aliquot of the analyte was injected in the splitless mode. We used helium as the carrier gas, a front inlet purge flow rate of 3 mL/min, and a gas flow rate of 1 mL/min through the column. The temperature was maintained at 50 °C for 1 min, then rose to 310 °C at a rate of 10 °C/min, and maintained at 310 °C for 8 min. The injection, transfer line, and ion source temperatures were 280 °C, 270 °C, and 220 °C, respectively. Energy was −70 eV in the electron impact mode. Mass spectrometry data were acquired in the full-scan mode with an m/z range of 50–500 at a rate of 20 spectra/second after a solvent delay of 460 s. Analyses were conducted on a minimum of five biological replicates per treatment.

We used Chroma TOF 4.3X software (LECO Corporation, Saint-Joseph, MI, USA) and the LECO-Fiehn Rtx5 database for raw peak extraction, data baseline filtering, baseline calibration, peak alignment, deconvolution analysis, peak identification, and integration of peak areas. The retention time index method was used for peak identification, with a tolerance of 5000.

### 2.4. sRNA Sequencing and Bioinformatic Analysis

Total RNA was separated using 15% denaturing PAGE to recover sRNAs 18–30 nt in length. The sRNAs were then purified and used to synthesize cDNA, which in turn was sequenced on the HiSeq 2500 platform. Known miRNAs were identified by the BLASTN search against the miRBase 20.0 database, using the default parameters. Potential novel miRNAs were identified using MIREAP software. We used the Web-based program psRNATarget to identify putative targets for known and novel miRNAs. Networks (conjoint analysis of miRNAs and mRNAs) were drawn using Cytoscape.

### 2.5. Hormone Quantification

Leaves from each treatment were used to analyze the levels of endogenous ABA and IAA. Extraction and purification were conducted following the methods described in [9]. Briefly, samples were ground into a powder with a mortar and pestle, and 100 mg subsamples were transferred to precooled 2 mL screw-cap tubes and stored in liquid nitrogen. We added 500 µL of extraction solvent (2-propanol:H_2_O:concentrated HCl (2:1:0.002, *v*/*v*/*v*)) to each tube, along with varying volumes of internal standard solutions. Tubes were centrifuged on a shaker at 100 rpm for 30 min at 4 °C. We then added 1 mL of dichloromethane to each sample and shook the samples for 30 min at 4 °C. Tubes were further centrifuged at 13,000× *g* for 5 min at 4 °C, and 900 µL solvent from the lower phase was transferred into a screw-cap vial and concentrated using a nitrogen evaporator. Samples were then redissolved in 100 μL of methanol, and 50 µL of sample solution was injected into a reverse-phase C18 Gemini HPLC column for high-performance liquid chromatography/electrospray ionization tandem mass spectrometry analysis. Quantitative analysis of hormones followed [17].

### 2.6. H_2_O_2_ Concentration

Fresh leaf samples (0.1 g) in 0.9 mL 50 mmol/L phosphate buffer (pH = 7.8) were homogenized in an ice bath and centrifuged at 10,000× *g* for 10 min at 4 °C. The concentration of H_2_O_2_ in the supernatant was determined colorimetrically using a commercial kit (BCA assay, Nanjing Jiancheng Bioengineering Institute, Nanjing, China). We used 3–5 leaves from three biological replicates per treatment to ensure that sufficient amounts of tissue were sampled.

### 2.7. Soluble Sugar, Soluble Protein, and Proline Concentrations

Three biological replicates per treatment were sampled for each of the following analyses. To assess the concentration of soluble sugar, 0.1 g of fresh leaves in 1 mL distilled water were homogenized in an ice bath and then placed in a water bath at 95 °C for 10 min. After cooling, the samples were centrifuged at 8000× *g* for 10 min at 25 °C and diluted with distilled water to 10 mL. The soluble sugar concentration in the supernatant was determined using anthrone colorimetry with a commercial kit (Suzhou Comin Bioengineering, Suzhou, China).

For the soluble protein concentration, 0.1 g of fresh leaves in 1 mL of distilled water were homogenized in an ice bath and then centrifuged at 8000× *g* for 10 min at 4 °C. The soluble protein concentration in the supernatant was determined by the Coomassie brilliant blue method using a commercial kit (Suzhou Comin Bioengineering).

To assess the proline concentration, 0.1 g of fresh leaves in 1 mL of distilled water were homogenized in an ice bath and then placed in a water bath at 90 °C for 10 min. After cooling, the samples were centrifuged at 10,000× *g* for 10 min at 25 °C. The proline concentration in the supernatant was determined by spectrophotometry using a commercial kit (Suzhou Comin Bioengineering).

### 2.8. Antioxidant Enzyme Activity Assays

Three biological replicates per treatment were sampled for the following analyses. Fresh leaf samples (0.1 g) in 1 mL 50 mmol/L phosphate buffer (pH 7.8) were homogenized in an ice bath and centrifuged at 8000× *g* for 10 min at 4 °C. Activities of SOD and CAT were assessed using commercial kits (Suzhou Comin Bioengineering).

SOD activity was evaluated by measuring the rate at which the enzymes inhibited O^2–^ production by xanthine morpholine with xanthine oxidase, as a function of the absorbance at 560 nm after 30 min. Measurements were conducted using a SOD assay kit. One unit of activity was defined as the quantity of SOD required to cause a 50% inhibition in the reduction of nitrotetrazolium blue chloride in 1 mL reaction solution.

CAT activity was measured by analyzing the decomposition rate of H_2_O_2_ expressed as a function of absorbance at 240 nm. One unit of enzyme was defined as the quantity required to break down 1 nmol of H_2_O_2_/min/mg tissue.

### 2.9. qRT-PCR

Total RNA was isolated from three biological replicates per treatment, as described above, for preparation and sequencing of the DEG library. Each RNA sample (approximately 1 μg of total RNA) was treated with gDNA Eraser (TaKaRa Bio Inc., Beijing, China) following the manufacturer instructions, to eliminate any contaminant gDNA. The treated RNA solution (10 μL) was reverse transcribed using the PrimeScript™ Reverse Transcriptase Reagent Kit with gDNA Eraser (Perfect Real Time; TaKaRa Bio Inc.) in accordance with the manufacturer’s protocols. Gene-specific primers were designed using Primer 5.0 and are listed in Table S5. *GAPDH* was used as the internal reference gene. qRT-PCR was performed on the Bio-Rad CFX96™ Real-Time System (Bio-Rad, Hercules, CA, USA) using the SYBR Premix Ex Taq™ Kit (Perfect Real Time; TaKaRa Bio Inc., Beijing, China) in accordance with the manufacturer’s protocols. qRT-PCR conditions were as follows: 30 s at 94 °C for denaturation, then 40 cycles of 5 s at 94 °C, 30 s at 56 °C, and 10 s at 72 °C. The relative expression levels of the target genes were calculated using the 2^−ΔΔCt^ comparative threshold cycle method. All reactions were conducted on three biological replicates, and the results were analyzed using Bio-Rad CFX Manager software (V1.6.541.1028).

### 2.10. Statistical Analysis

The experimental data between control and treatments was analyzed using one-way ANOVA followed by Tukey’s post-hoc test with a significance level of 0.05 (*p* < 0.05; SPSS 18.0 software for Windows; SPSS, Chicago, IL, USA) [18].

## 3. Results

### 3.1. Physiological and Biochemical Changes

**Figure 1:**
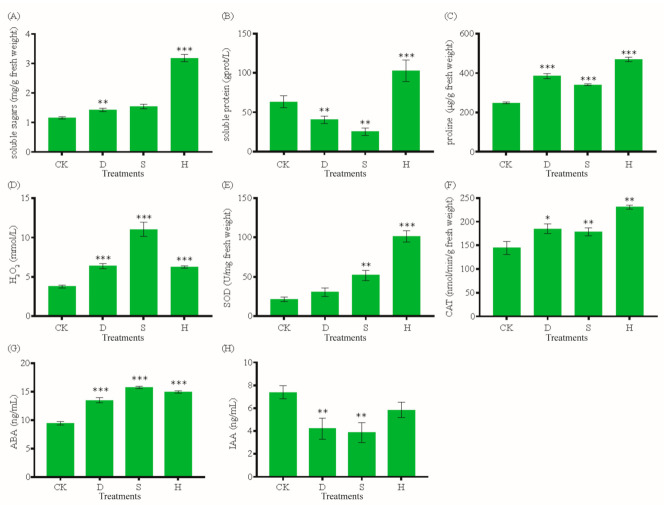
Physiological and biochemical characteristics of *G. biloba* under stress treatments. (**A**) Soluble sugars, (**B**) soluble proteins, (**C**) proline, (**D**) H_2_O_2_, (**E**) SOD, (**F**) CAT, (**G**) ABA, and (**H**) IAA. Error bars represent the mean ± SD; *n* = 3; */**/***, *p* < 0.05/0.01/0.001, respectively, compared with CK. CK: Control; D: Drought; S: Salt; H: Heat; SOD: Superoxide dismutase; CAT: Catalase; ABA: Abscisic acid; IAA: Indole-3-acetic acid.

Drought (D), salt (S), and heat (H) stresses significantly affected the physiological and biochemical statuses of leaves. We examined the effects of D, S, and H on the concentrations of soluble proteins, soluble sugars, proline, and H_2_O_2_ and the activities of antioxidant enzymes (SOD and CAT), ABA, and IAA in leaves. The soluble sugar concentration increased significantly under H (Figure 1A). The soluble protein concentration decreased by 36% under D and 60% under S, but increased by 62% under H (Figure 1B). By contrast, the concentrations of both proline and H_2_O_2_ increased significantly under all stress treatments (Figure 1C,D). SOD activity was higher under S and H, and CAT activity increased under all stress treatments (Figure 1E,F). The leaf ABA concentration also increased under all stress treatments (Figure 1G), whereas IAA decreased by 42.84%, 47.71%, and 20.93% under D, S, and H, respectively (Figure 1H).

### 3.2. Changes in Metabolites

We determined changes in metabolites under D, S, and H using gas chromatography–time-of-flight–mass spectrometry (GC–TOF–MS). We identified 168 metabolites from seven categories, including carbohydrates, amino acids, lipids, organic acids, phenols, flavonoids, and others (Table S1). Next, using principal component analysis (Figure S2A, Table S2) and orthogonal projection to latent structure with discriminant analysis (Table S3), we classified metabolites by treatment group based on high-dimensional spectral measurements using GC–MS. Metabolites differed among treatments (a variable important in projection >1 and *p* < 0.05). The metabolome view map indicates that pathways enriched under D compared with the control group (CK; *p* < 0.05) included those involved in biosynthesis of valine, leucine, and isoleucine and metabolism of glycine, serine, and threonine (Figure S2B). Compared with CK, different metabolites involved in beta-alanine metabolism and the pentose phosphate pathway were enriched under S (Figure S2C), whereas metabolites associated with arginine and proline metabolism and the pentose phosphate pathway were enriched under H (Figure S2D).

**Figure 2:**
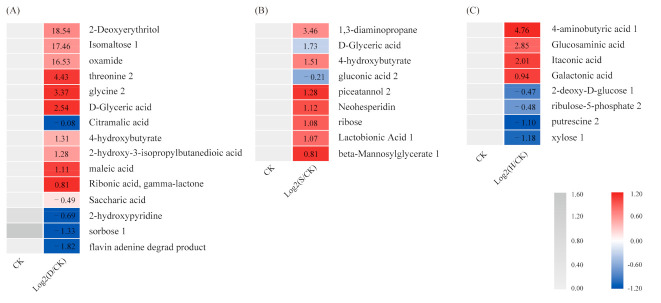
Heatmap of metabolites. (**A**) D vs. CK, (**B**) S vs. CK, and (**C**) H vs. CK. D: Drought; S: Salt; H: Heat; CK: Control.

The major metabolites that accumulated under the stress treatments are listed in Table S1. The metabolite heat map (Figure 2) indicated that isomaltose 1, oxamide, threonine 2, glycine 2, D-glyceric acid, and 4-hydroxybutyrate all increased significantly under D compared with CK, whereas sorbose 1 decreased (Figure 2A). Piceatannol 2, D-glyceric acid, 1, 3-diaminopropane, 4-hydroxybutyrate, and lactobionic acid 1 all increased significantly under S (Figure 2B). In addition, 4-aminobutyric acid 1, glucosaminic acid, and galactonic acid all increased significantly under H, whereas putrescine 2 and xylose 1 decreased (Figure 2C).

### 3.3. Differentially Expressed Genes

We generated RNA-Seq data from leaves and obtained clean reads for three biological replicates from each treatment (Table S4). We used differential expression gene (DEG) analysis to examine the transcriptomic responses to D, S, and H (Supporting Information Figure S3). We filtered 397, 1007, and 4494 DEGs under D, S, and H, respectively (Figure S3A–C). Patterns in the heat map indicate that gene expression trends under D and S were similar but were significantly different from those under H (Figure S3D).

### 3.4. Respiratory Metabolism

**Figure 3:**
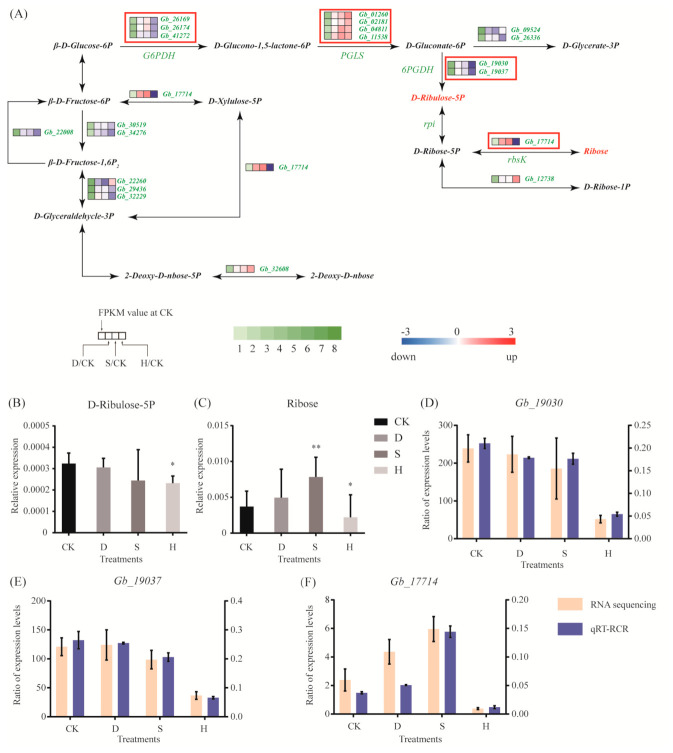
Gene expression and metabolites involved in respiratory metabolism. (**A**) DEGs associated with the respiratory pathway. Red and blue indicate upregulation and downregulation, respectively. Red frames indicate the upregulated or downregulated genes discussed in the main text. (**B**,**C**) Expression of (**B**) D-ribose-5P and (**C**) ribose. Error bars represent the mean ± SD; *n* = 5; */**, *p* < 0.05/0.01, respectively, compared with CK. (**D**–**F**) Expression of (**D**) *Gb_19030* (6PGDH), (**E**) *Gb_19037* (6PGDH), and (**F**) *Gb_17714* (*rbsK*), as determined by qRT-PCR. Error bars represent the mean ± SD; *n* = 3. CK: Control; D: Drought; S: Salt; H: Heat.

We analyzed the differential expression of genes that are key to the regulation of respiratory metabolism. In the pentose phosphate pathway, expression of PGLS, which regulates D-glucono-1,5-lactone-6P, hydrolyzed to D-gluconate-6P was upregulated under all three stress treatments, such as *Gb_01260*, *Gb_02181*, *Gb_04811*, and *Gb_11538* (Figure 3A). However, expression of three genes (*Gb_26169*, *Gb_26174*, and *Gb_41272*) encoding glucose-6-phosphate dehydrogenase (G6PDH) and two genes (*Gb_19030* and *Gb_19037*) encoding 6-phosphogluconate dehydrogenase (6PGDH) decreased significantly under H, but not under D or S. In addition, *Gb_17714*, which is involved in the metabolic link between D-ribose-5P and ribose, was upregulated under S but downregulated under H (Figure 3A). This is consistent with the metabolomic results, in which ribose content increased under S but D-ribulose-5P and ribose contents decreased under H (Figure 3B,C). In addition, we used quantitative reverse-transcription PCR (qRT-PCR) to verify key genes and confirmed that *Gb_19030*, *Gb_19037*, and *Gb_17714* were downregulated under H (Figure 3D–F), whereas *Gb_17714* was upregulated under S (Figure 3F).

### 3.5. Amino Acid Metabolism

**Figure 4:**
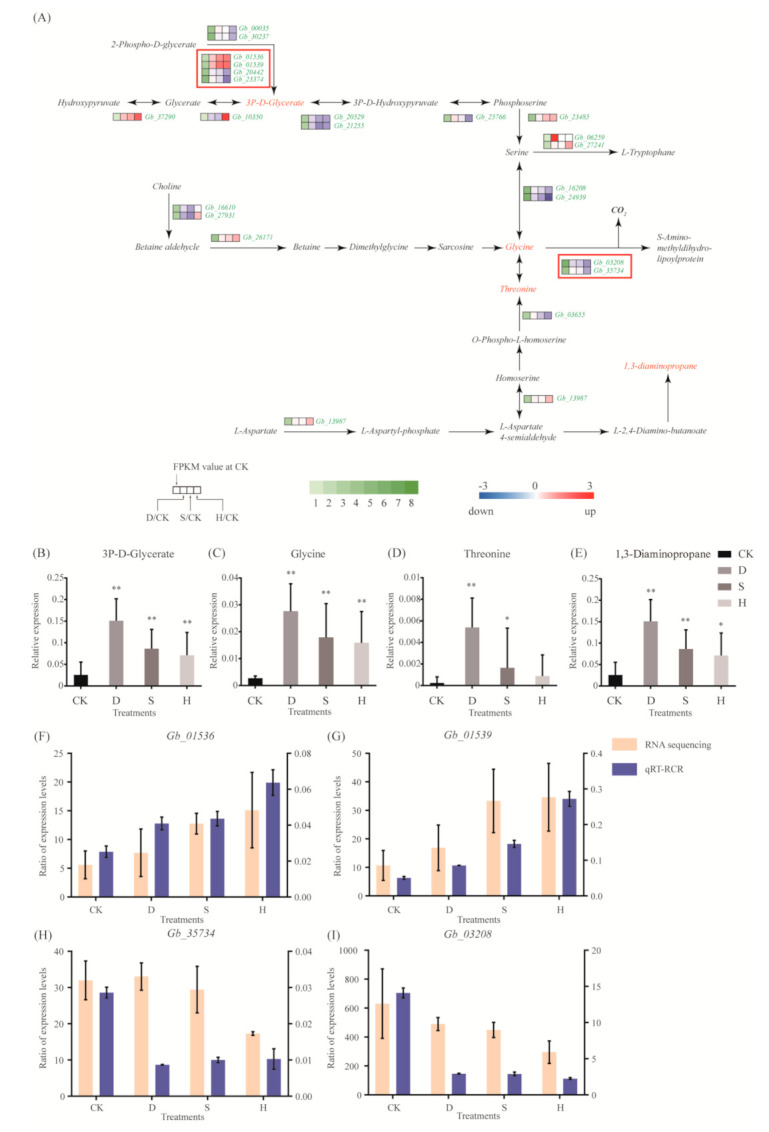
Gene expression and metabolites involved in the glycine, serine, and threonine metabolic pathways. (**A**) DEGs associated with these pathways. Red and blue indicate upregulation and downregulation, respectively. Red frames indicate the upregulated or downregulated genes discussed in the main text. (**B**–**D**) Expression of (**B**) 3P-D-glycerate, (**C**) glycine, (**D**) threonine, and (**E**) 1,3-diaminopropane. Error bars represent the mean ± SD; *n* = 5; */**, *p* < 0.05/0.01, respectively, compared with CK. (**F**–**I**) Expression of (**F**) *Gb_01536*, (**G**) *Gb_01539*, (H) *Gb_35734,* and (I) *Gb_03208*, as determined by qRT-PCR. Error bars represent the mean ± SD; *n* = 3. CK: Control; D: Drought; S: Salt; H: Heat.

Most genes in the glycine, serine, and threonine metabolic pathway, including *Gb_01536*, *Gb_01539*, *Gb_37290*, *Gb_23485*, *Gb_06259*, and *Gb_27241*, were upregulated in response to all three treatments (Figure 4A). Two genes, *Gb_03208* and *Gb_35734*, which are involved in the transformation of glycine into S-amino-methyldihydro-lipoylprotein and CO_2_, were downregulated under H (Figure 4A). Results of metabolomic analyses indicate that 3P-d-glycerate, glycine, threonine, and 1,3-diaminopropane levels increased in response to all three stress treatments (Figure 4B–E). Results of qRT-PCR confirmed the upregulation of *Gb_01536* and *Gb_01539* (Figure 4F,G) and the downregulation of *Gb_35734* and *Gb_03208* (Figure 4H,I).

**Figure 5:**
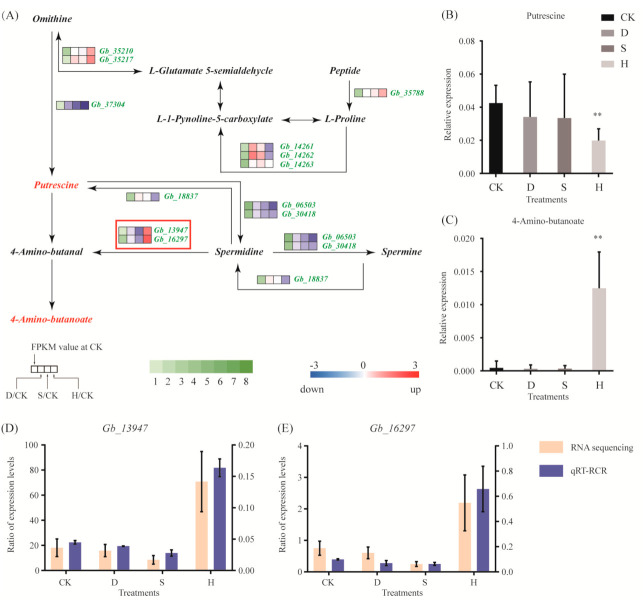
Gene expression and metabolites involved in the arginine and proline metabolic pathways. (**A**) DEGs associated with these pathways. Red and blue indicate upregulation and downregulation, respectively. Red frames indicate the upregulated or downregulated genes discussed in the main text. (**B**,**C**) Expression of (**B**) putrescine and (**C**) 4-amino-butanoate. Error bars represent the mean ± SD; *n* = 5; **, *p* < 0.01, compared with CK. (**D**,**E**) Expression of (**D**) *Gb_13947* and (**E**) *Gb_16297*, as determined by qRT-PCR. Error bars represent the mean ± SD; *n* = 3. CK: Control; D: Drought; S: Salt; H: Heat.

Few genes in the arginine and proline metabolic pathway exhibited significant changes in response to D or S; however, H triggered upregulation of *Gb_13947* and *Gb_16297*, which were involved in the synthesis of 4-amino-butanoate (Figure 5A). The qRT-PCR results confirmed these results (Figure 5D). In addition, putrescine decreased (Figure 5B) and 4-amino-butanoate increased in response to H (Figure 5C).

### 3.6. Key Genes Responding to Drought, Salt, and Heat Stresses

**Figure 6:**
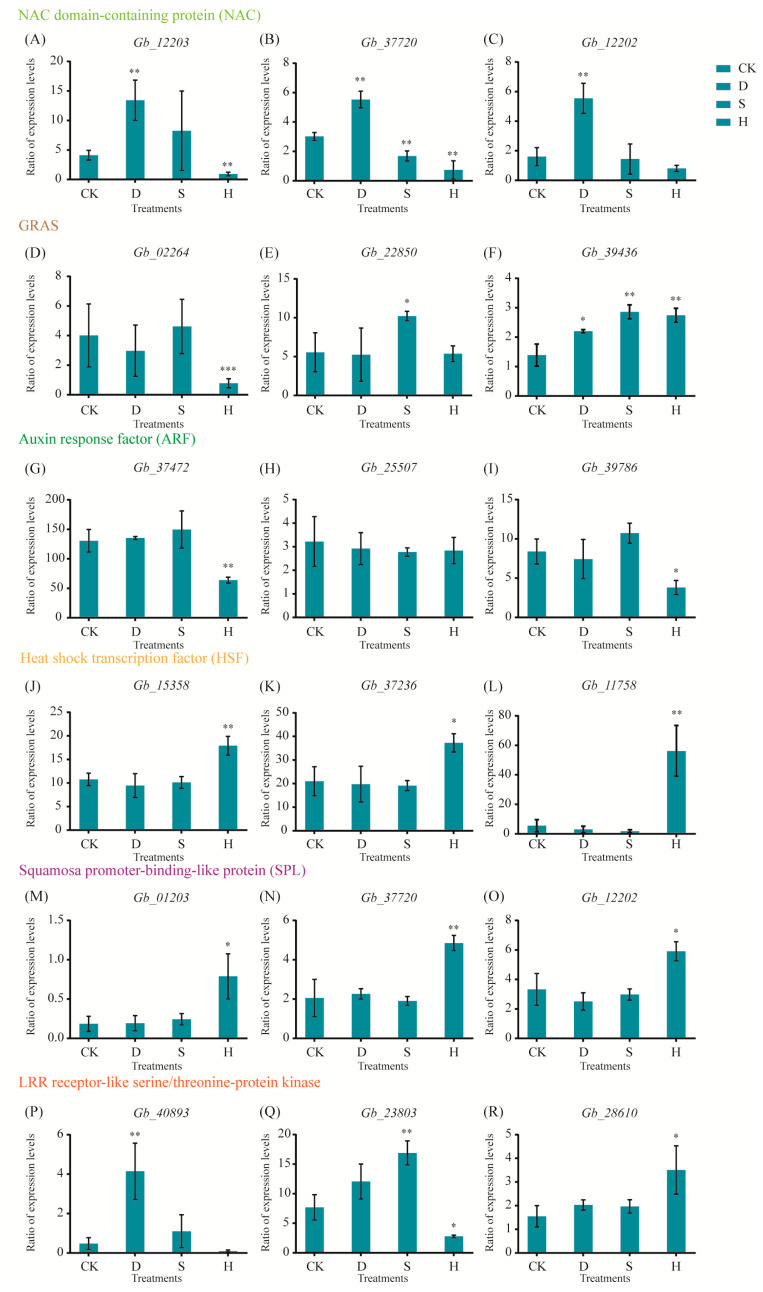
Expression of key genes under drought, salt, and heat stresses based on qRT-PCR. (**A**–**C**) NAC genes: (**A**) *Gb_12203*, (**B**) *Gb_37720*, and (**C**) *Gb_12202.* (**D**–**F**) GRAS genes: (**D**) *Gb_02264*, (**E**) *Gb_22850*, and (**F**) *Gb_39436.* (**G**–**I**) ARF genes: (**G**) *Gb_37472*, (**H**) *Gb_25507*, and (**I**) *Gb_39786.* (**J**–**L**) HSF genes: (**J**) *Gb_15358*, (**K**) *Gb_37236*, and (**L**) *Gb_11758.* (**M**–**O**) SPL genes: (**M**) *Gb_01203*, (**N**) *Gb_37720*, and (**O**) *Gb_12202.* (**P**–**R**) LRR receptor-like serine/threonine-protein kinase genes: (**P**) *Gb_40893*, (**Q**) *Gb_23803*, and (**R**) *Gb_28610.* Error bars represent the mean ± SD; *n* = 3; */**, *p* < 0.05/0.01, respectively, compared with CK. CK: Control; D: Drought; S: Salt; H: Heat.

We performed transcriptome sequencing to identify critical genes in responses to drought, salt, and heat; these genes included nitrogen assimilation control (NAC), GRAS, auxin response factor (ARF), heat shock transcription factors, squamosa promoter-binding-like protein (SPL), and leucine-rich repeat (LRR) receptor-like serine/threonine protein kinase. NAC genes (*Gb_12203*, *Gb_37720*, and *Gb_12202*) were upregulated under D but downregulated or unaffected in response to S and H (Figure 6A–C). Genes related to GRAS responded differently to the three treatments; for example, *Gb_02264* was downregulated under H, *Gb_22850* was upregulated under S, and *Gb_39436* was upregulated under all three treatments (Figure 6D–F). Under H, genes related to ARF (*Gb_37472* and *Gb_39786*) were downregulated, whereas *Gb_25507* was not significantly affected (Figure 6G–I). Similarly, genes related to HSF (*Gb_15358*, *Gb_37236*, and *Gb_11758*; Figure 6J–L) and SPL (*Gb_01203*, *Gb_37720*, and *Gb_12202*) were upregulated under H (Figure 6M–O). Genes encoding LRR receptor-like serine/threonine protein kinase exhibited different responses to the three treatments; for example, *Gb_40893* was upregulated only in response to D, *Gb_23803* only to S, and *Gb_28610* only to H (Figure 6P–R).

### 3.7. Small RNAs and Corresponding Target Genes

**Figure 7:**
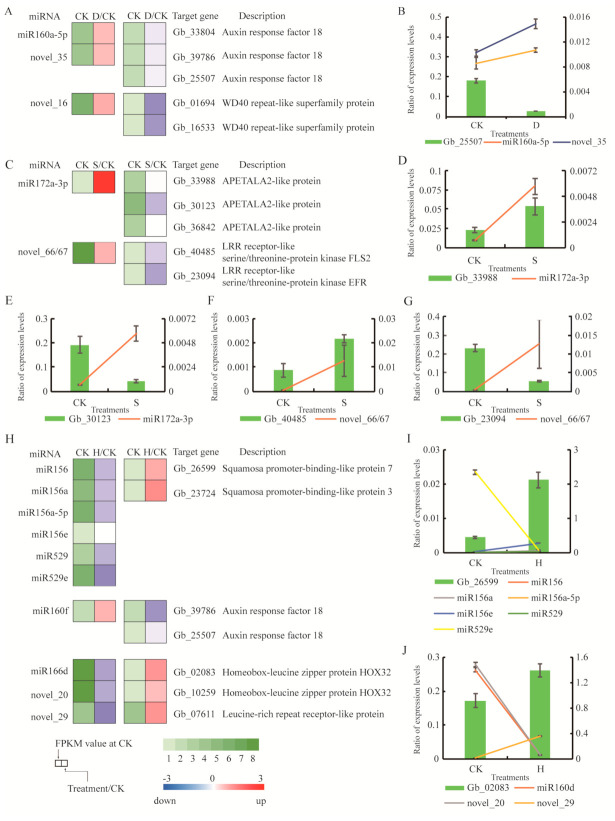
Expression of small RNAs and their corresponding targets in response to stress treatments. (**A**) Differential expression of miRNAs and their target genes under drought. (**B**) qRT-PCR analysis of miR160a, novel_35, and their target genes under drought stress. (**C**) Differential expression of miRNAs and their target genes under salt stress. (**D**,**E**) qRT-PCR analysis of miR172a-3p and their target genes under salt stress. (**F**,**G**) qRT-PCR analysis of novel_66/67 and their target genes under salt stress. (**H**) Differential expression of miRNAs and their target genes under heat stress. (**I**) qRT-PCR analysis of miR156, miR156a, miR156a-5p, miR156e, miR529, miR529e, and their target genes under heat stress. (**J**) qRT-PCR analysis of miR160d, novel_20, novel_29, and their target genes under heat stress. CK: Control; D: Drought; S: Salt; H: Heat.

We sequenced small RNA (sRNA) libraries for all treatments to identify miRNAs involved in stress responses. Known miRNAs were annotated by aligning them to sequences from all plant species included in miRBase. Functional annotation was used to evaluate the potential functions of target genes. We selected several miRNA/target gene pairs from the three libraries and investigated their expression patterns using qRT-PCR. Under D, genes involved in ARF (*Gb_33804*, *Gb_39786*, and *Gb_25507*) were predicted targets of miR160a-5p, and *Gb_01694* and *Gb_16533* (WD40 repeat-like superfamily protein) were predicted targets of novel_35 and novel_16 (Figure 7A). These miRNAs were upregulated under D, whereas expression of their target genes was downregulated (Figure 7A). The expression profiles of miR160a-5p, novel_35, and their target genes were confirmed. The trend exhibited by *Gb_25507* was the opposite of that exhibited by miR160a-5p and novel_35 (Figure 7B). Under S, *Gb_33988*, *Gb_30123*, and *Gb_36842*, all of which are related to APETALA2-like protein (AP2), were predicted targets of miR172a-3p, whereas *Gb_40485* and *Gb_23094* (LRR receptor-like serine/threonine-protein kinase, FLS2) were predicted targets of novel_66/67 (Figure 7C). These miRNAs were upregulated under S, whereas their target genes were downregulated (Figure 7C). Expression profiles of miR172a-3p, novel_66/67, and their target genes exhibited contrary trends in two pairs: *Gb_30123* with miR172a-3p (Figure 7E), and *Gb_23094* with novel_66/67 (Figure 7G). Under H, genes related to SPL (*Gb_26599* and *Gb_23724*) were predicted targets of miR156, miR156a-5p, miR156e, miR529, and miR529e, whereas genes encoding leucine zipper proteins (*Gb_02083*, *Gb_10259*, and *Gb_07611*) were predicted targets of miR166d, novel_20, and novel_29 (Figure 7H). These miRNAs were downregulated in response to H, and expression of their target genes was upregulated (Figure 7H). The expression profiles of miR156, miR156a, miR156a-5p, miR156e, miR529, miR529e, miR166d, novel_20, novel_29, and their target genes exhibited opposite trends in two pairs: *Gb_26599* with miR529e (Figure 7I) and *Gb_02083* with miR160d and novel_20 (Figure 7J).

## 4. Discussion

### 4.1. Physiological and Biochemical Responses

Plant hormone levels typically vary upon exposure to strong light, high temperatures, salt, and drought. ABA and IAA are important signaling molecules that regulate metabolic processes related to stress adaptation and induce stress resistance in plants [19]. Numerous studies have shown that the ABA content increases under abiotic stress and induces the expression of downstream genes [20]. We found that leaf ABA content increased significantly under drought, salt, and heat stresses, while IAA content decreased significantly under drought and salt stresses. Given that stomatal closure is correlated with ABA accumulation, *G. biloba* leaves may regulate stomatal closure via ABA accumulation to maintain water content under conditions of osmotic stress associated with drought and salt, thereby reducing damage from osmotic stress and improving tolerance to drought and salt. In addition, increased ABA content induced upregulated expression of many downstream transcription factors and genes, thereby activating downstream metabolic pathways. IAA content decreased under drought and salt stresses, potentially inhibiting growth and thereby relieving the pressure that osmotic stress places on normal physiological activities.

Environmental stress results in excessive ROS production in plants. Due to their high activity and toxicity, ROS increase the susceptibility of cells to oxidative stress. Plants have an efficient system of antioxidant defenses, involving both enzymes (SOD, CAT, POD, APX, and GPX) and other compounds (ascorbic acid, glutathione, and alkaloids). These help control and eliminate ROS, thus protecting plant cells from oxidative damage [21]. Numerous studies have demonstrated that the antioxidant defense system is essential for cell protection during drought, salt, or heat stress [22]. SOD and CAT activities increased significantly under drought and salt stresses [12,23]. We found that H_2_O_2_ content in *G. biloba* leaves increased significantly under all three stress types, implying that the stress treatments induced ROS accumulation. Correspondingly, the increased activities of antioxidant enzymes, including SOD and CAT, imply that these enzymes may mitigate ROS damage. These results are similar to those of liquorice under salt and drought stresses [12] and chickpea under salt stress [23]. However, the degree to which SOD activity increased varied among the treatments; whereas SOD activity increased significantly under salt stress, the increase under heat stress was much more pronounced, suggesting that plants may respond to different stresses and scavenge excess ROS via different antioxidant enzymes.

Osmotic regulation is the physiological mechanism that allows plants to adapt to drought or salt stress and enhance their resistance to such stress. Proline is an osmotic regulatory solute, ROS scavenger, and molecular chaperone that stabilizes protein structure, thus protecting cells from stress-induced damage [24]. Under osmotic stress, most plants accumulate osmotic regulatory substances to maintain osmotic balance in cells, prevent cell dehydration, and protect the structure and function of biological macromolecules. For example, proline content increased significantly in poplar at 45 °C [25]. Soluble sugar and protein may also play important roles in osmotic stress [26]. In the study, contents of soluble sugar and proline increased significantly in response to all stress treatments, and soluble protein accumulated significantly under heat stress. These results are consistent with changes of the soluble sugar and protein in *Arabidopsis* under heat stress [26]. Our results indicate that *G. biloba* may synthesize large amounts of soluble sugars, proteins, and proline to improve stress tolerance, and that these compounds may protect the membrane system and maintain cell stability. Our study was based on the short-term treatments (24 h) of the mechanism to response to heat, drought, and salt stresses in *G. biloba*. However, longer term (a week, a month, etc.) stress responses are still unclear.

### 4.2. Key Metabolic Pathways and Metabolites

The pentose phosphate pathway is important for respiratory metabolism in plants, producing NADPH, and providing reducing power for biosynthesis of carbohydrates. The pentose phosphate pathway also plays a role in the responses to environmental stress. G6PDH and 6PGDH are key rate-limiting enzymes in the pentose phosphate pathway, controlling the metabolic rate of the pathway as a whole. Expression of G6PDH increased significantly in wheat under high salt stress [27], whereas it exhibited a downward trend in *Arabidopsis* under salt stress, osmotic stress, and low temperatures [28]. In rice, the expression of 6PGDH was upregulated under salt, PEG, and ABA treatments [29,30], whereas the expression of G6PDH exhibited no significant changes under high salinity or low temperatures [30]. We found that the expression of G6PDH genes (*Gb_26169*, *Gb_26174*, and *Gb_41272*) and 6PGDH genes (*Gb_19030* and *Gb_19037*) was downregulated under heat stress but was not significantly affected by drought or salt stress. However, the expression trends of G6PDH and 6PGDH genes were significantly changed in wheat [27], *Arabidopsis* [28], and rice [30] under salt stress, indicating the different response mechanism compared with *G. biloba*. Three genes (*Gb_19030*, *Gb_19037*, and *Gb_17714*) involved in the regulation of 5-ribulose-5p and ribose were downregulated under heat stress, but *Gb_17714* was upregulated under drought and salt stresses. In addition, the results of the metabolomic analysis indicated that ribulose 5-phosphate and ribulose were downregulated under heat stress. These results indicate that the pentose phosphate pathway may be inhibited under heat stress, but not under drought or salt stress, suggesting different response pathways under heat stress compared with drought and salt stresses.

Amino acids substantially affect plant growth and development via their roles in physiological metabolism. Amino acids, or substances synthesized with amino acids as precursors, may improve the adaptability of plants to adversity and stress. Glycine is an important amino acid and a synthetic substrate of glycine betaine [31], which in turn plays an active role in stress tolerance [32]. Aminobutyric acid, a non-protein amino acid, is a regulatory substance synthesized by glutamate, which may enhance the activities of peroxidase, CAT, and other antioxidant enzymes, thus reducing damage caused by peroxidation. High levels of 4-aminobutyric acid (GABA) may accumulate under a variety of adverse environmental conditions [33]. GABA metabolism is related to carbon–nitrogen metabolism balance and ROS scavenging and enhances the structural stability of the cell membrane while reducing damage to the photosynthetic system [34]. Stress treatments upregulated numerous genes involved in the glycine, serine, and threonine metabolic pathways, including *Gb_01536*, *Gb_01539*, *Gb_37290*, *Gb_23485*, *Gb_06259*, and *Gb_27241*; by contrast, genes involved in the degradation pathway, including *Gb_03208* and *Gb_*35734, were downregulated, potentially promoting accumulation of glycine and threonine. Given that glycine and threonine may act as synthetic substrates of osmotic regulatory substances, accumulation of glycine and threonine plays a critical role in the response of *G. biloba* to abiotic stress. In addition, the putrescine content decreased, whereas GABA content increased significantly, in the arginine and proline metabolic pathway under heat stress, suggesting that accumulation of GABA may activate antioxidant defense reactions, thereby mitigating oxidative and heat damage by increasing osmotic regulatory substances.

### 4.3. Key Transcription Factors and Genes

Most plant species contain specific transcription factors and genes that respond to different stress conditions. NAC is a plant-specific transcription factor that plays a critical role in the response to abiotic stress. In *Arabidopsis*, genes encoding NAC transcription factors participate in the responses to drought, salt, and cold stress [35,36]. Similarly, in rice, *OsNAC5* and *OsNAC6* are involved in the responses to drought, high salinity, and low temperatures [37,38]. In addition, expression of *TaNAC69* increases in wheat under drought stress [39]. In *G. biloba*, the expression of NAC transcription factor genes *Gb_12203*, *Gb_37720*, and *Gb_12202* were significantly upregulated under drought, but were either significantly downregulated or unaffected under salt and heat stresses, indicating that these transcription factors are involved mainly in the drought response in *G. biloba*.

GRAS transcription factors are plant-specific transcription factors involved in the regulation of various biological processes, including growth and development, hormone interactions, and responses to biotic and abiotic stresses [40]. GRAS transcription factors are related to the development of roots and buds, which often suffer under abiotic stress. Previous studies have demonstrated that some GRAS transcription factors play a positive regulatory role in plant responses to abiotic stress. For example, in *Arabidopsis*, the GRAS protein SCL14 plays an important role in activating stress-induced promoters [41]. NtGRAS1 may increase ROS levels under different stress treatments in tobacco [42]. OsGRAS23 was also reported to be a positive regulator in rice [43]. The SlGRAS40 protein regulates IAA and gibberellin signaling pathways in tomato to mitigate the effects of abiotic stress during growth and development [44]. We observed significant upregulation of the GRAS transcription factor gene *Gb_22850* under salt stress, while another GRAS gene (*Gb_39436*) was significantly upregulated in response to all three stress treatments, suggesting that *Gb_22850* is involved predominantly in the response to salt stress, whereas *Gb_39436* responds to a broader range of abiotic stressors.

HSFs are important for the response to heat stress. Reviews have summarized the transcriptional and post-transcriptional regulatory networks of heat stress responses [3,45]. HSFs are important in signal transduction cascades, activating heat-induced response genes and serving as key transcription factors in the network regulating heat-induced responses [3]. In *Arabidopsis*, HSFA1s was shown to play a central role in the heat stress response and therefore is generally regarded as a master regulator critical to the activation of transcriptional networks for the response to heat stress [46,47]. Many important heat-induced transcription factors, such as DREB2A, HSFA2, HSFA7a, and HSFBs, are thought to be regulated directly by HSFA1s [47]. We identified three HSF genes in *G. biloba*, including *Gb_15358* (homologous with *HSFA1*), *Gb_37236* (homologous with *NnHSF24*), and *Gb_11758* (homologous with *GrHSF8*). These transcription factors were upregulated only under heat stress and did not respond significantly to drought or salt stress. Therefore, they may be important regulators of the response pathways to heat stress.

Receptor kinase proteins (RLKs) are located on plant cell membranes and can sense external signals and stimuli [48]. LRR receptor-like kinases are the largest subgroup of receptor kinases [49], playing an important role in the responses to biotic and abiotic stresses [50]. For example, LRR-RPK1 (*RPK1*) may enhance tolerance to drought and oxidative stress in *Arabidopsis* [51], while LRR receptor-like kinases may enhance drought tolerance in rice [52] and cold tolerance in wild soybean [53]. We identified numerous LRR genes in *G. biloba* that promote resistance to various stresses and contribute to the long life span of the species [10]. Some of these genes may be significantly upregulated in response to abiotic stresses, including *Gb_40893* (drought stress), *Gb_23803* (salt stress), and *Gb_28610* (heat stress). These results suggest that certain LRR genes may be specific to the responses to particular stressors.

### 4.4. Key miRNAs and Functions of Target Genes

miRNAs regulate the metabolic processes of growth and development and are involved in the responses to abiotic stresses such as drought, salt, and heat [54,55,56]. For example, miR160 targets the ARF gene and participates in the IAA signal transduction pathway, hormone homeostasis, and plant morphogenesis. ARFs regulate the expression of IAA-induced genes by binding to IAA promoters. High or low expression of *ARF10* can lead to severe developmental defects [57]. ARF2 is involved in the ABA signaling pathway, and ARF2 mutants exhibit high sensitivity to exogenous ABA [58]. In tomato under drought stress, miR160 targets ARF10 to regulate the ABA signaling pathway, maintain water balance in leaves, and ensure normal development [59]. We found that miR160a and novel_35 targeted three genes involved in the IAA signaling pathway. Expression of miR160a and novel_35 was upregulated under drought stress, while their corresponding target genes were downregulated. This negative feedback pattern was confirmed by qRT-PCR. An increased miR160a level inhibited the expression of its target gene, ARF, under drought stress, suggesting its involvement in the IAA signaling pathway. In addition, decreased IAA content in leaves under drought stress may slow growth rates, thereby mitigating the stress of water deficiency and alleviating the damage caused by dehydration.

Many plant species exhibit miRNA-mediated salt stress response networks. Studies have identified numerous miRNAs involved in salt stress responses in corn [60], rice [61], wheat [62,63], barley [64], and sugarcane [65]. AP2/ERF, the target gene of miR172, is part of an important family of transcription factors involved in the responses to biotic and abiotic stresses. For example, AP2/ERF (RAP2.6) in *Arabidopsis* is involved in the abiotic stress response via ABA-dependent pathways [66]. We found that miR172a targets three AP2 genes, *Gb_33988*, *Gb_30123*, and *Gb_36842*, which exhibit a negative feedback pattern under salt stress. qRT-PCR further identified *Gb_30123* (apetala2-like protein) as a target of miR172a. Given that ABA content increased significantly under salt stress, these results suggest that miR172a may participate in the response to salt stress in *G. biloba* by inhibiting expression of *Gb_30123* and thereby improving salt resistance.

Numerous miRNAs are also involved in the heat shock regulation network [45]. Studies have reported that miRNAs exhibit varying responses to abiotic stress. For example, miR166 may be induced and upregulated under drought stress in alfalfa, wheat, and barley [67,68], but it may also be downregulated under salt and cold stresses in maize and rice [60,69]. HD-zip, the target gene of miR166, plays an important role in the response to osmotic stress. In *Arabidopsis*, a drought- and ABA-induced HD-zip, *AThb-6/7/12*, was upregulated in response to dehydration [70]. We found that miR166d, novel_20, and novel_29 targeted three homeobox-leucine zipper protein genes, *Gb_02083*, *Gb_10259*, and *Gb_07611*, which exhibited opposite expression patterns under heat stress. Furthermore, we found that the expression of miR166d, novel_20, and their target gene *Gb_02083* (homeobox-leucine zipper protein, HOX32) exhibited an opposite regulatory trend. Heat stress may result in water loss in *G. biloba* leaves, leading to dehydration. HD-zip may be involved in dehydration responses; thus, our results suggest that miR166d and novel_20 participate in the response to osmotic stress via regulation of HOX32.

## 5. Conclusions

**Figure 8:**
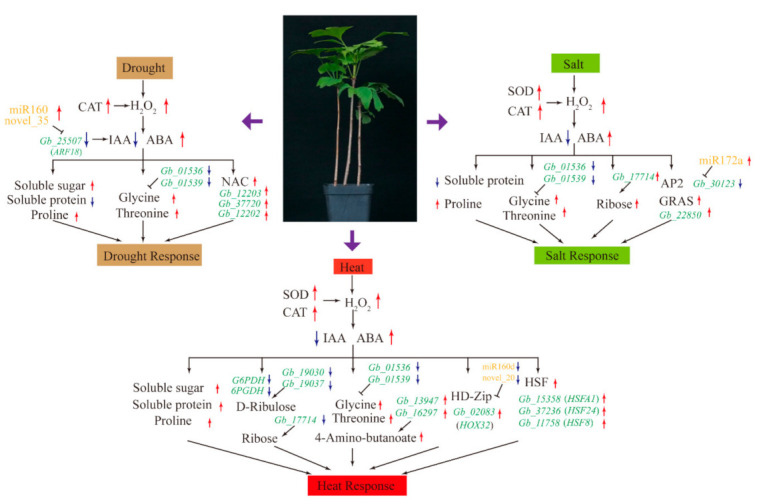
Schematic diagram of stress responses, showing major changes in physiology, biochemistry, metabolism, and transcription. Red and blue represent upregulation and downregulation, respectively. CAT: Catalase; SOD: Superoxide dismutase; ABA: Abscisic acid; IAA: Indole-3-acetic acid; NAC: NAC transcription factor; HSF: Heat shock transcription factor.

We provided detailed information on the multilevel responses of *G. biloba* to drought, salt, and heat stresses (Figure 8). *G. biloba* responds to drought stress primarily by increasing CAT activity, ROS production, the concentrations of ABA, soluble sugar, proline, glycine, and threonine, and the expression of miR160 and NAC. Under salt stress, we observed increased activities of SOD and CAT, increased concentrations of ABA, ROS, proline, glycine, threonine, and ribose, and increased expression of miRNA172a and GRAS. Under heat stress, in addition to increases in the concentrations of antioxidant enzymes, ABA, ROS, soluble sugar, proline, glycine, and threonine, we observed increases in soluble protein content and expression of HD-Zip and HSFs.

## Supplementary Materials

The following are available online at https://www.mdpi.com/2218-273X/10/12/1635/s1, Figure S1: Two-year-old seedlings of *G. biloba*, Figure S2: Metabolomic analyses and changes in metabolite levels in the leaves of G. biloba under the stress treatments, Figure S3: Analysis of DEGs under stress treatments, Table S1: Major metabolites accumulated under stress treatments, Table S2: Accumulation interpretation rate of principal component analysis model, Table S3: Accumulation interpretation rate of the orthogonal projection to latent structure with discriminant analysis model, Table S4: Summary statistics of sequencing data quality and mapping to the reference genome, Table S5: Primer sequences used in qRT-PCR.
