# Supplementary material for: Physiological, Transcriptomic, and Metabolic Responses of Ginkgo biloba L. to Drought, Salt, and Heat Stresses
Source: Biomolecules. 2020 Dec 3;10(12):1635. doi: 10.3390/biom10121635 (PMC7761781; doi:10.3390/biom10121635)
Supplement: Supplementary file 1 [file biomolecules-10-01635-s001.pdf]

Figure S1

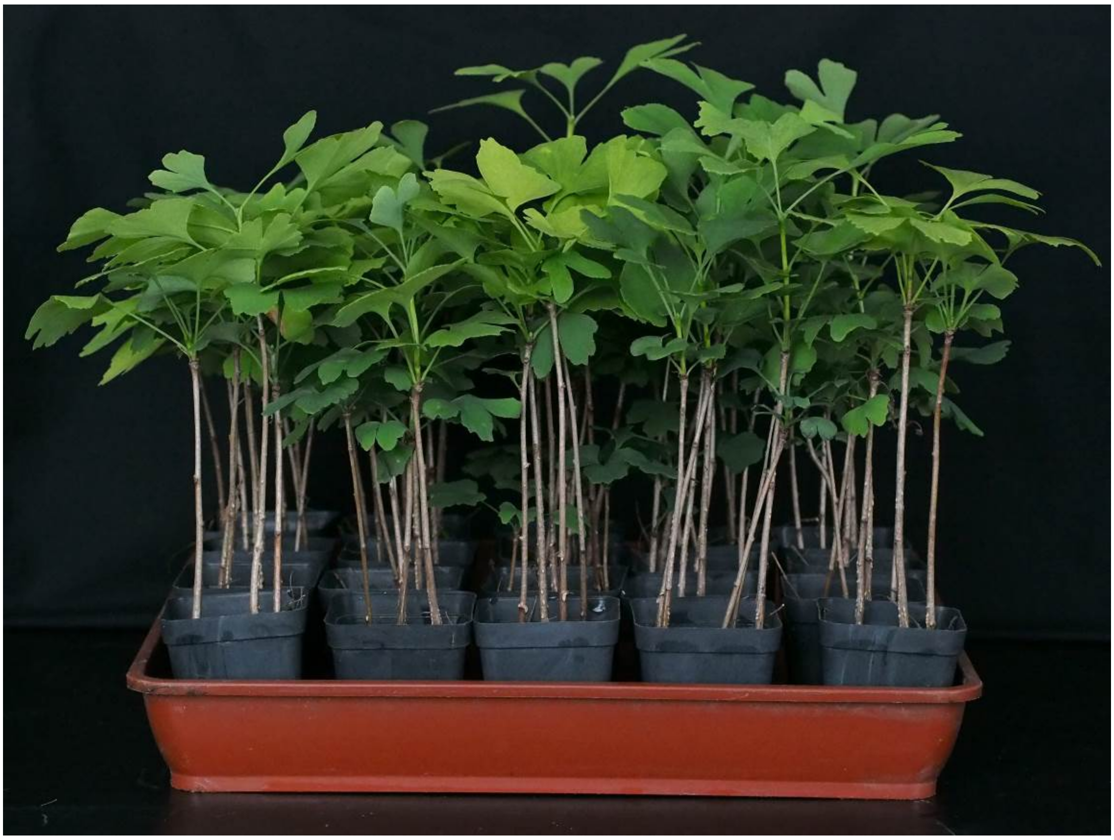

Figure S1 Two-year-old seedlings of *G. biloba*

Figure S2

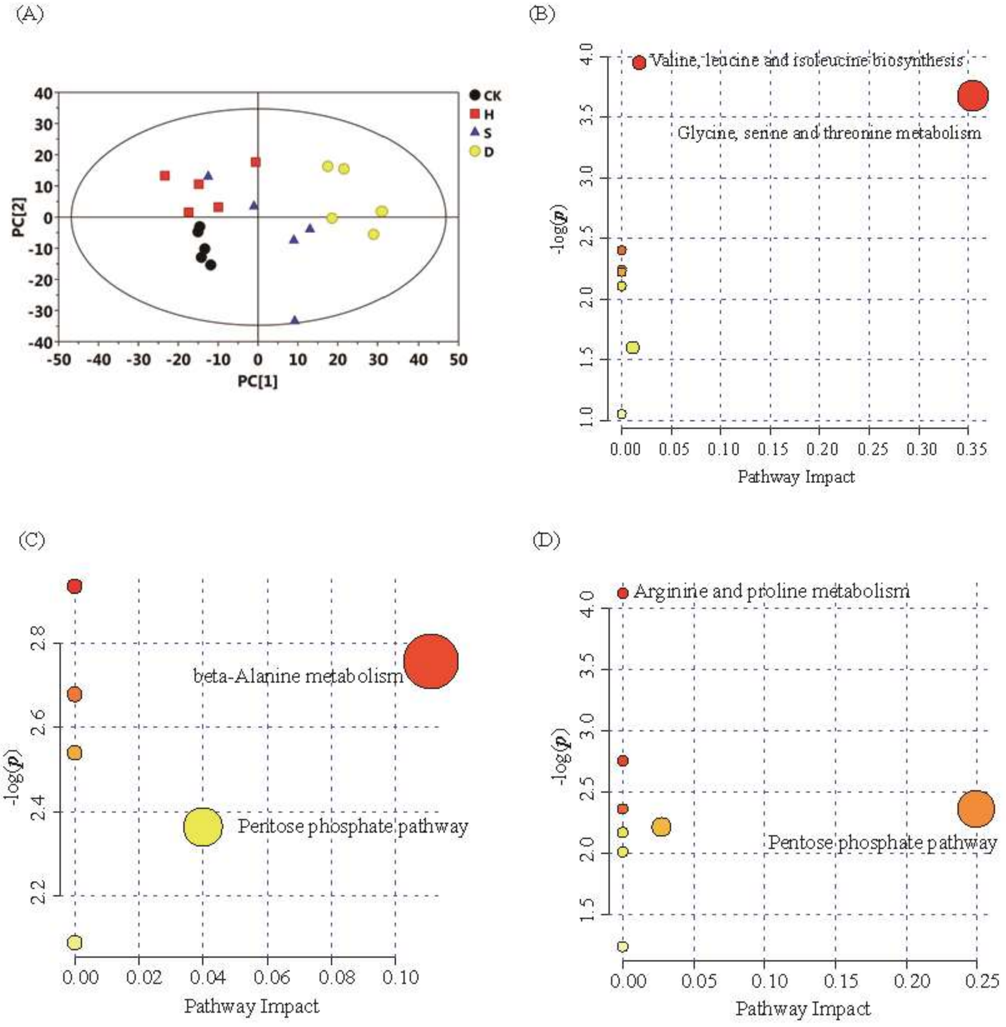

Figure S2. Metabolomic analyses and changes in metabolite levels in the leaves of *G. biloba* under the stress treatments. (A) Principal component analysis plot. Black circle = CK, red square = H, blue triangle = S, yellow circle = D. (B)–(D) Pathway enrichment of different metabolites (B) D vs. CK, (C) S vs. CK. and (D) H vs. CK. Each treatment was represented by five biological replicates. Circle color represents the p-value, whereas circle size is in proportion to the number of metabolites involved.

Figure S3

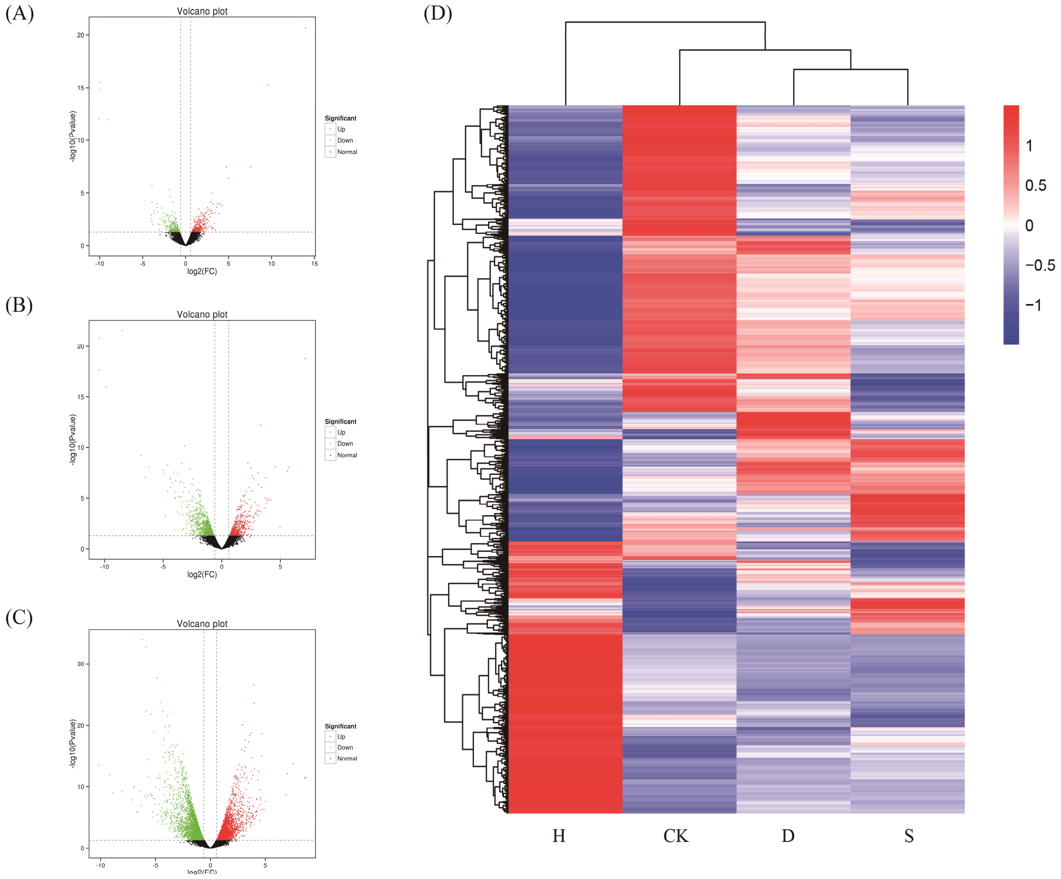

Figure S3 Analysis of DEGs under stress treatments. (A)–(C) Volcano plots of DEGs under (A) drought, (B) salt stress, and (C) heat stress. (D) Heatmap of DEGs under stress treatments. Red and blue indicate upregulation and downregulation, respectively.

Table S1 Major metabolites accumulated under stress treatments

Metabolites	mean CK	Log2(D/CK)	Log2(S/CK)	Log2(H/CK)
**Carbohydrate**				
2-deoxy-D-glucose 1	0.110	0.213	-0.230	-0.469
2-Deoxyerythritol	0.000	18.535	16.845	-0.078
3,6-Anhydro-D-galactose 4	0.014	0.367	0.197	-0.299
6-deoxy-D-glucose 1	0.003	-18.245	-0.568	1.131
arbutin	0.005	0.108	0.653	-0.125
cellobiose 1	0.000	-13.953	2.103	0.092
Cellobiotol	0.001	1.056	0.853	0.864
D-Altrose 2	0.003	-0.836	0.144	-1.553
d-Glucoheptose 1	0.003	-0.197	0.188	0.651
Digitoxose 1	0.016	0.678	0.538	0.454
D-Talose 2	0.007	-0.939	-0.121	0.805
Erythrose 2	0.020	0.339	0.664	-0.145
fructose 2	0.047	-0.715	-0.080	0.005
fructose-6-phosphate	0.000	0.265	0.199	-1.383
fucose 1	0.001	-5.763	-17.031	0.470
Galactinol 3	0.008	-0.268	0.153	-1.038
galactose 2	0.432	-0.067	-0.105	-0.036
Gentiobiose 1	0.018	-0.468	0.076	-0.730
Glucose-1-phosphate	0.049	0.318	0.436	0.419
glucose-6-phosphate 1	0.015	-0.008	0.215	0.367
Isomaltose 1	0.000	17.463	17.130	-0.078
Isopropyl-beta-D-thiogalactopyranoside	0.003	0.008	0.334	0.557
lactose 2	0.000	16.091	14.886	-0.078
lactulose 2	0.004	0.090	-0.180	-0.061
Levoglucosan	0.004	0.374	0.084	-1.603
L-Threose 1	0.000	2.285	1.941	0.840
Lyxose 1	0.001	-0.009	-0.464	-0.601
maltose	0.009	0.290	1.138	-0.152
maltotriose 1	0.006	-1.158	-0.332	-18.817
mannose 2	0.560	0.130	0.126	0.575
melibiose 2	0.006	-0.039	-0.310	-0.020
Palatinose	0.019	-0.517	-0.196	-0.533
ribose	0.004	0.418	1.076	-0.749
ribulose-5-phosphate 2	0.000	-0.085	-0.410	-0.478
salicin	0.002	0.037	-0.372	0.648
Sophorose 2	0.010	-0.888	-0.468	-0.662
sorbose 1	1.516	-1.333	-0.096	-0.120
sucrose	0.013	-0.751	-0.667	-1.027
Tagatose 1	0.102	-0.170	-0.562	-0.004
trehalose-6-phosphate	0.014	0.389	-0.010	1.149
Sucrose-6-Phosphate	0.230	-0.424	0.569	0.780
Turanose 1	0.000	0.215	-1.339	0.006
uridine 1	0.047	-1.206	-0.101	-0.273
xylose 1	0.016	-0.019	-0.524	-1.183
**Amino acids**				

3-Hydroxynorvaline 2	0.001	0.941	0.433	0.587
alanine 1	0.007	-0.162	0.739	-0.110
beta-Alanine 2	0.002	0.376	0.409	-0.169
canavanine 1	0.000	-0.065	-0.147	-0.753
N-Methyl-DL-alanine	0.001	0.287	-0.351	-0.868
serine 2	0.003	1.702	0.764	0.705
threonine 2	0.000	4.429	2.717	1.809
valine	0.005	-0.585	0.519	0.032
**Lipid**				
11-beta-prostaglandin-F-2-alpha 2	0.000	0.999	1.099	0.233
D-erythronolactone 2	0.001	0.133	-0.194	0.194
Gluconic lactone 1	0.006	-0.318	0.463	-0.285
Linoleic acid methyl ester	0.006	1.001	1.362	0.430
Methyl Palmitoleate	0.001	-16.144	-0.184	-1.325
Ribonic acid, gamma-lactone	0.006	0.809	0.131	-0.030
**Organic acid**				
(2R,3S)-2-hydroxy-3-	0.000	0.488	1.126	0.660
2,3-Dimethylsuccinic acid	0.001	0.174	0.212	-0.270
2-hydroxy-3-isopropylbutanedioic acid	0.001	1.283	0.736	1.296
2-hydroxybutanoic acid	0.000	-14.273	-0.562	0.452
2-Methylglutaric Acid	0.002	0.786	0.884	0.740
3-Aminoisobutyric acid 1	0.002	0.185	0.525	-0.227
3-Hexenedioic acid	0.001	0.336	0.268	0.070
3-hydroxybenzoic acid	0.002	-0.290	-0.945	0.139
1-Aminocyclopropanecarboxylic acid	0.005	0.061	-0.764	-0.687
3-Hydroxypropionic acid 1	0.000	1.785	1.077	-13.666
4-aminobutyric acid 1	0.000	-0.722	-0.449	4.761
4-Hydroxybenzoic acid	0.004	0.007	-0.296	-0.058
4-hydroxybutyrate	0.001	1.313	1.507	0.114
4-hydroxycinnamic acid	0.017	0.608	0.284	-0.327
6-hydroxy caproic acid	0.001	0.617	0.870	0.725
6-phosphogluconic acid	0.000	1.100	1.210	0.599
Aminomalonic acid	0.001	1.261	1.313	0.233
arachidonic acid	0.010	-0.405	-0.350	-0.467
beta-Mannosylglycerate 1	0.005	0.073	0.812	0.294
Capric Acid	0.001	1.390	0.962	1.456
Chlorogenic Acid 2	0.000	-0.410	-0.133	-0.128
Citraconic acid 2	0.003	0.410	0.694	0.577
Citramalic acid	0.000	-0.077	0.787	0.704
citric acid	0.000	1.323	-2.913	-14.077
D-galacturonic acid 1	0.001	-0.027	-1.347	-0.498
D-Glyceric acid	0.026	2.537	1.730	1.450
Galactonic acid	0.000	0.146	0.486	0.940
Gallic acid	0.004	-0.601	-0.467	-0.993
Glucoheptonic acid 2	0.004	0.109	-1.206	-1.513
gluconic acid 2	0.000	15.911	-0.214	-0.078
Glucosaminic acid	0.000	2.384	1.282	2.855
glucuronic acid 2	0.001	1.164	1.369	0.959
Glutaric Acid	0.001	0.047	0.648	0.218

glycine 2	0.003	3.369	2.741	2.571
glycolic acid	0.020	-0.236	-0.171	0.158
histidine 1	0.000	-0.483	23.562	-0.078
isocitric acid 2	0.090	-0.813	-0.651	-0.311
Isoleucine	0.001	-0.493	0.906	-0.407
Itaconic acid	0.001	1.415	0.977	2.010
lactic acid	0.014	-0.691	-0.790	0.278
Lactobionic Acid 1	0.003	0.233	1.074	0.848
L-Allothreonine 1	0.003	-0.368	-0.862	-1.245
linoleic acid	0.001	-0.586	-0.444	-1.547
L-Malic acid	0.307	-0.076	-0.069	-0.021
maleic acid	0.002	1.109	0.542	0.120
malonic acid 1	0.000	14.279	13.781	14.280
mucic acid	0.001	-0.201	-0.382	-0.504
oxalacetic acid	0.000	0.262	1.228	1.867
palmitic acid	0.002	0.349	0.555	0.334
prostaglandin A2 3	0.000	-0.304	-1.151	-1.892
Pyrrole-2-Carboxylic Acid	0.004	-18.862	-18.593	-18.457
Pyruvic acid	0.004	-0.606	1.244	0.669
quinic acid	0.037	-0.359	0.226	-0.041
Saccharic acid	0.001	-0.492	-0.552	-0.455
shikimic acid	0.003	0.110	-1.676	-18.140
stearic acid	0.002	-0.254	0.131	-0.537
succinic acid	0.027	1.119	0.917	0.541
sulfuric acid	0.015	-0.036	0.736	0.777
tartaric acid	0.003	-0.099	0.017	0.069
Threonic acid	0.005	0.578	0.224	0.273
trans-3-hydroxycinnamic acid	0.001	-1.179	-0.316	-15.976
urocanic acid 2	0.000	18.873	17.582	-0.078
**Phenols**				
(+)-catechin	0.000	16.984	18.104	15.392
1,2,4-Benzenetriol	0.002	0.100	0.181	0.133
catechol	0.000	1.124	1.509	1.217
phloroglucinol	0.000	-0.204	-1.418	-0.185
pyrogallol	0.001	0.307	0.444	0.270
Thymol	0.000	1.309	1.106	0.357
resorcinol	0.003	0.361	0.300	-0.720
**Flavonoid**				
chrysin	0.000	-0.099	0.449	-0.733
Epicatechin	0.009	-1.207	-0.950	-0.715
Epigallocatechin	0.005	0.365	-0.036	-0.282
Neohesperidin	0.074	0.346	1.123	-0.574
Tricetin	0.001	-0.007	0.307	0.297
**Others**				
1,3-diaminopropane	0.000	2.341	3.463	3.392
2,3-Dihydroxypyridine	0.001	0.303	0.448	-0.088
2-hydroxypyridine	0.653	-0.695	-0.395	-0.418
3-Hydroxypyridine	0.026	-0.736	-0.707	-0.728
4-Androsten-11beta-ol-3,17-dione 1	0.000	-0.252	-0.356	-0.474

4-HYDROXYPYRIDINE	0.006	-0.075	0.382	-0.467
7,8-Dimethylalloxazine	0.000	-0.318	0.482	0.054
Aldosterone 2	0.000	0.247	-0.773	0.074
ascorbate	0.023	0.015	-0.549	-0.283
Biuret 3	0.000	-13.158	1.370	0.617
conduritol b epoxide 2	0.009	0.102	1.408	1.461
creatine degr	0.022	0.269	0.038	0.106
D-Arabitol	0.013	-0.839	0.002	0.083
D-erythro-sphingosine 1	0.007	-0.389	0.048	-0.235
DL-Anabasine 1	0.003	0.876	0.935	0.340
Ethanolamine	0.014	1.430	0.377	0.760
flavin adenine degrad product	0.013	-1.821	0.290	0.709
fumaric acid	0.004	1.013	0.969	1.015
glutaraldehyde 3	0.000	16.366	15.936	-0.078
glycerol	0.053	1.154	0.433	0.832
inosine	0.000	-0.327	0.914	1.806
Lactamide 2	0.257	-1.531	-1.196	-0.449
Maleamate 4	0.000	-1.840	-0.218	-0.536
myo-inositol	8.083	-0.313	-0.117	-0.327
N-Acetyl-beta-D-mannosamine 4	0.013	-1.099	0.419	0.169
naringenin 2	0.147	-1.119	0.127	-0.599
N-cyclohexylformamide 2	0.009	0.367	0.613	-0.505
oxamide	0.000	16.526	16.422	-0.078
oxoproline	0.045	0.566	0.350	-1.342
palatinitol 2	0.002	-0.851	0.044	0.185
Phenyl beta-D-glucopyranoside	0.015	0.626	0.543	-0.478
phosphate	0.692	-0.397	-0.725	-1.105
phytosphingosine 2	0.003	0.105	0.791	-0.116
piceatannol 2	0.001	0.122	1.276	0.467
putrescine 2	0.043	-0.317	-0.346	-1.096
Threitol	0.005	1.063	0.847	0.804

Table S2 Accumulation interpretation rate of principal component analysis model

Model	Type	A	N	R^2X(cum)	Q^2 (cum)	Title
M1	PCA-X	3	20	0.278	-0.1	TOTAL
M2	PCA-X	2	10	0.297	-0.21	CK-H
M3	PCA-X	2	10	0.313	-0.2	CK-S
M4	PCA-X	2	10	0.372	-0.0482	CK-D
M5	PCA-X	2	10	0.304	-0.172	H-S
M6	PCA-X	2	10	0.349	-0.0831	H-D
M7	PCA-X	2	10	0.3	-0.189	S-D

Table S3 Accumulation interpretation rate of the orthogonal projection to latent structure with discriminant analysis model

Model	Type	A	N	R^2X(cum)	R^2Y(cum)	Q^2 (cum)	Title
M1	OPLS-DA	1+1+0	10	0.296	0.988	0.336	CK-H
M2	OPLS-DA	1+1+0	10	0.311	0.997	0.478	CK-S
M3	OPLS-DA	1+1+0	10	0.349	0.999	0.735	CK-D
M4	OPLS-DA	1+1+0	10	0.281	0.992	0.344	H-S
M5	OPLS-DA	1+1+0	10	0.306	0.998	0.671	H-D
M6	OPLS-DA	1+1+0	10	0.274	0.996	0.388	S-D

Table S4 Summary statistics of sequencing data quality and mapping to the reference genome

Samples	Base Number	Clean Reads	Mapped Reads	GC Content	%≥Q30	Mapped Ratio
CK1	10,558,488,736	35,650,767	25,676,290	46.24%	92.35%	72.02%
CK2	9,025,040,374	30,708,446	22,039,370	47.38%	91.93%	71.77%
CK3	6,169,879,150	20,865,173	15,487,506	45.71%	92.22%	74.23%
D1	7,086,998,684	23,992,059	18,039,332	45.52%	92.06%	75.19%
D2	7,083,227,548	24,037,590	17,987,805	46.74%	91.79%	74.83%
D3	8,000,180,018	27,090,867	20,407,482	45.62%	92.13%	75.33%
S1	8,108,064,596	27,489,140	21,141,110	46.47%	92.46%	76.91%
S2	10,093,181,990	35,036,589	25,738,279	46.24%	92.66%	73.46%
S3	6,759,302,830	22,775,604	17,951,378	45.92%	91.56%	78.82%
H1	8,028,032,574	27,145,571	20,249,403	45.95%	92.23%	74.60%
H2	8,941,508,044	30,429,320	19,704,904	45.54%	92.55%	64.76%
H3	6,739,217,658	22,916,027	16,242,648	45.98%	92.41%	70.88%

Table S5 Primer sequences used in qRT-PCR

Gene ID	primer(5’-3’)
GAPDH	F: CTGCCAAGGCTGTAGGTAAGG<br>R: TCAGATTCCTCCTTGATGGCG
Gb_19030	F: TTGTCTACCAAATATCCCTCACCTT<br>R: TGACAAACCCAGAGCTACACG
Gb_19037	F: AGTCTGCATGGCGTAGAGTTGTG<br>R: GTGTGGAAGGAGCCAGGCATATC
Gb_17714	F: TTCTCCAAGTCGCACTGGTTCAAG<br>R: TGTCTGCTACTTCGCCTACTCCTC
Gb_01536	F: TGGCGGTGGTGGTGCTAGAC<br>R: GGTTGGAGTGTTGGCGACGTG
Gb_01539	F: CTCCTGGATCGCTTATGCTTGGTC<br>R: GAACCGACACCGGAAGAAGAAGAG
Gb_35734	F: AAGGAACACCAGCAGGAAGTCAAC<br>R: GGCAAGTCCGACAGGTCCAATG
Gb_03208	F: CATGCCAGGTCTCGTGTTCCATC<br>R: TCAGAAGGTGTCGGCGAATTGTG
Gb_13947	F: CCGACGCATTGAGCAACAGTTC<br>R: GTGCCTCGTGACGCCAATAGG
Gb_16297	F: GGCTATTCTGGTTCCAAGGTGGTG<br>R: CGTGCCTCGTGACGCCAATAG
Gb_25507	F: ACGCAGAGTGACGCCAACAATG<br>R: CTGAACTTCCACACCTCGCCATG
Gb_33988	F: AGCTTCGTCAAGTCAGGCAATCG<br>R: GCTTGCTGCTACCAGGCTGTG
Gb_30123	F: ACCTCAGCCTACTTCCTTCTCAGG<br>R: GCGATACTGCGAACTCCTGGAAC
Gb_40485	F: TTGGAACGGTGCCAGTGGAATTG<br>R: AAGTGGTATTGCTGCCGCTAACG
Gb_23094	F: TGTGGATTGAGCTTGAGTGAACGG<br>R: ATGTGCAGTCGTATTGTCGTCCAG
Gb_26599	F: TCAGCAGTGCAGCAGATTCCATG<br>R: TCTTCCGACGGCGTTCATTGTG
Gb_02083	F: CGGCTGGAACTCGAACATCTGG<br>R: TGCGACAACACTACGGACATACTG
miR160a	F: TGCCTGGCTCCCTGTATGC

miR172a-3p	F: CCGCAGAATCTTGATGATGCTGCAT
miR156	F: CGCTGACAGAAGAGAGTGAGCAC
miR156a	F: GCTGACAGAAGAGAGTGAGCACA
miR156a-5p	F: GCGTGACAGAAGAGAGTGAGCAC
miR156e	F: CGCTTGACAGAAGATAGAGGGCAC
miR529	F: CCGAGAAGAGAGAGAGCACAGCTT
miR529e	F: GCAGAAGAGAGAGAGTACAGCCC
miR160d	F: TCGGACCAGGCTTCATTCCC
novel_20	F: TCGGACCAGGCTTCATTCCC
novel_29	F: ACGCTCTTCTCTCTCGGCTTG
novel_35	F: TGCCTGGCTCCCTGTATGC
novel_66/67	F: CCGCTCCTTTTGTGGAAACTCTTCC
